# Myofibroblasts and lung fibrosis induced by carbon nanotube exposure

**DOI:** 10.1186/s12989-016-0172-2

**Published:** 2016-11-04

**Authors:** Jie Dong, Qiang Ma

**Affiliations:** Receptor Biology Laboratory, Toxicology and Molecular Biology Branch, Health Effects Laboratory Division, National Institute for Occupational Safety and Health, Centers for Disease Control and Prevention, 1095 Willowdale Road, Morgantown, WV USA

**Keywords:** Carbon nanotube, Myofibroblast, Lung fibrosis, Animal model, Mechanism, Extracellular matrix

## Abstract

Carbon nanotubes (CNTs) are newly developed materials with unique properties and a range of industrial and commercial applications. A rapid expansion in the production of CNT materials may increase the risk of human exposure to CNTs. Studies in rodents have shown that certain forms of CNTs are potent fibrogenic inducers in the lungs to cause interstitial, bronchial, and pleural fibrosis characterized by the excessive deposition of collagen fibers and the scarring of involved tissues. The cellular and molecular basis underlying the fibrotic response to CNT exposure remains poorly understood. Myofibroblasts are a major type of effector cells in organ fibrosis that secrete copious amounts of extracellular matrix proteins and signaling molecules to drive fibrosis. Myofibroblasts also mediate the mechano-regulation of fibrotic matrix remodeling via contraction of their stress fibers. Recent studies reveal that exposure to CNTs induces the differentiation of myofibroblasts from fibroblasts in vitro and stimulates pulmonary accumulation and activation of myofibroblasts in vivo. Moreover, mechanistic analyses provide insights into the molecular underpinnings of myofibroblast differentiation and function induced by CNTs in the lungs.

In view of the apparent fibrogenic activity of CNTs and the emerging role of myofibroblasts in the development of organ fibrosis, we discuss recent findings on CNT-induced lung fibrosis with emphasis on the role of myofibroblasts in the pathologic development of lung fibrosis. Particular attention is given to the formation and activation of myofibroblasts upon CNT exposure and the possible mechanisms by which CNTs regulate the function and dynamics of myofibroblasts in the lungs. It is evident that a fundamental understanding of the myofibroblast and its function and regulation in lung fibrosis will have a major influence on the future research on the pulmonary response to nano exposure, particle and fiber-induced pneumoconiosis, and other human lung fibrosing diseases.

## Background

Mammalian lungs are among the most susceptible organs to fibrosis [1]. As the primary respiratory organ, the lungs perform gas exchange between the blood and the inhaled air through a thin layer of alveolar septal structures. These structures are vulnerable to structural and functional alterations, such as interstitial thickening and alveolar destruction, major pathologic features of lung fibrosis. Respiration also exposes the lungs constantly to numerous inhaled fibrogenic agents including toxic chemicals, mycobacteria, and particulate matters [2, 3]. Some chemicals are preferentially uptaken by lung epithelial cells and therefore tend to accumulate in the lungs upon exposure through either respiration or systemic means [4–6]. These exposures can damage the lung tissue to give rise to induced fibrosis. Lung fibrosis also occurs as a common, end stage pathologic development of existing lung diseases caused by infection, chronic inflammation, cardiovascular malfunction, autoimmunity, and idiopathy [7]. In many cases, human lung fibrosis is progressive and refractory to therapy, causing high rates of mortality and disability [8].

The progressive nature and severe outcome of human lung fibrosing diseases are well illustrated by idiopathic pulmonary fibrosis (IPF). IPF initiates insidiously with no known etiology and follows a chronic but progressive course that is ultimately lethal, indicated by the median survival of IPF patients within 2–5 years after diagnosis [9–11]. IPF exhibits a histopathologic pattern of usual interstitial pneumonia, including the occurrence of mild-to-moderate inflammatory infiltration, injury and hyperplasia of alveolar epithelial cells, excessive deposition of the extracellular matrix (ECM), thickening of the alveolar septa, scarring and formation of fibroblastic foci, and temporally heterogeneous fibrotic remodeling of the lung structure, such as honeycombing [9, 11]. No effective therapy is available to the patients with this destructive lung disease except lung transplantation at the present time. Several animal models have been developed and studied for lung fibrosis such as IPF during the past few decades [12, 13]. Among these, the bleomycin-induced lung fibrosis is the most commonly used model. However, bleomycin-induced lung fibrosis differs from IPF in several pathologic aspects. First, bleomycin induces severe alveolar epithelial cell death in the lungs before the onset of fibrosis, whereas extensive alveolar epithelial cell lesions are rarely seen in IPF. Second, bleomycin causes diffusive fibrosis throughout the lung parenchyma with scattered fibrotic foci, while the fibrosis in IPF is predominantly in the form of fibroblastic foci. Third, fibrosis in the bleomycin mouse model resolves spontaneously and thus is reversible, but that in IPF is progressive and irreversible. Overall, the research using the bleomycin and other fibrosis models has not yielded effective translation to the treatment of IPF and other human lung fibrosing diseases including pneumoconiosis caused by exposure to fibrogenic particles and fibers, such as silica and asbestos [4, 14]. As a result, there are increasing efforts to gain improved fundamental understanding of the pathogenesis and development of lung fibrosis, as well as renewed interests in identifying new animal models that mimic human lung fibrosis, in order to achieve better treatment and prevention against human lung fibrosing diseases [8].

Carbon nanotubes (CNTs) are long and hollow nanostructures made of a single layer or concentric multiple layers of one-atom-thick carbon walls, designated as single-walled CNTs (SWCNTs) and multi-walled CNTs (MWCNTs), respectively [15]. As new materials, CNTs have been developed with a variety of industrial and commercial applications for electronic, biomedical, and energy-related uses. The annual production of CNTs has been increased rapidly in recent years [16]. On the other hand, most CNTs are respirable fibers with physicochemical features like nano-scaled diameter, fiber-like shape (high aspect ratio), large surface area, poor solubility, and excessive biopersistence, properties often associated with the fibrogenic and tumorigenic activities of inhaled particles and fibers, thus raising concern over the potential adverse health effects of human exposure to CNTs [17, 18]. Indeed, a marked progress in the understanding of CNT toxicity has been achieved in experimental animal and cell systems during the past decade. Importantly, the research has identified CNTs as a significant fibrogenic inducer in the lungs and the pleural space to cause interstitial, bronchial, and pleural fibrosis in animals [18–27]. The pathologic development and features of CNT-induced pulmonary interstitial fibrosis overlap with those of IPF and pneumoconiosis considerably. From the experimental research point of view, this finding suggests a possible application of CNT-induced lung fibrosis in the study of the human lung fibrosing diseases with regard to their pathogenesis, therapeutic targeting, and biomarkers for exposure and disease monitoring [18, 24, 28]. Recent field studies on CNT-exposed populations demonstrate marked accumulation of inflammatory and fibrotic mediators and biomarkers in the body fluids of workers manufacturing MWCNTs, highlighting a need to protect humans from nano exposure from occupational, environmental, and commercial sources [29–31].

The mechanisms by which CNTs cause lung fibrosis remain unclear, but are believed to involve an exacerbated fibroblastic response [18]^1^. During fibrosis development, fibroblasts and myofibroblasts act as major effector cells to produce excessive amounts of collagen fibers and other ECM proteins, and to remodel and contract the fibrosing tissues [32–40]. The fibroblastic response in fibrosis bears certain similarity to wound healing following tissue injury. At the early stage of lung fibrosis, resident fibroblasts in the lung interstitial space are activated upon stimulation. Activated fibroblasts migrate and proliferate to result in the accumulation of active fibroblastic cells in regions where injury takes place. At the same time, activated fibroblasts differentiate into myofibroblasts, which are a group of multi-functional mesenchymal cells implicated in wound healing, organ fibrosis, tumorigenesis, and cancer metastasis. Myofibroblasts are characterized by their simultaneous presentation of a high capacity of ECM protein production and their smooth muscle cell-like contractile features obtained through the *de novo* synthesis of α-smooth muscle actin (α-SMA)-containing stress fibers [34]. In physiologic wound healing, excessive ECM production and remodeling are avoided, as the majority of the α-SMA-expressing myofibroblasts disappear by way of apoptosis upon scar formation. However, during pathologic fibrosis, myofibroblasts become resistant to apoptosis and thereby persist to continuously synthesize and remodel the ECM, which ultimately leads to organ fibrosis and destruction [41]. Understanding the formation, function, and fate of myofibroblasts in tissue remodeling may hold a key to differentiating between physiologic wound healing and the development of organ fibrosis including IPF, pneumoconiosis, and CNT-induced lung pathology.

Several excellent reviews have been published to sum up the biological effects, the overall mode of action, and the interrelations between the physicochemical properties and the bioactivities of CNTs from a toxicological point of view [17–19, 42]. However, the cellular and molecular basis underlying the fibrotic response to CNTs, which is key to understanding the adverse health effects from CNT exposure, remains a topic of considerable challenge. In part, this issue is due to a lack of significant mechanistic insights into organ fibrosis in general. Given the emerging and rapidly advancing research on myofibroblasts and their function in the pathogenesis of fibrosis, we discuss the current literature on CNT-induced rodent lung fibrosis with focus on the formation and role of myofibroblasts in the development of pulmonary fibrosis. Possible mechanisms by which CNTs regulate myofibroblast functions and dynamics during fibrosis development in the lungs will be discussed. We anticipate that such analysis will have a major impact on the future research of the pulmonary response to CNT exposure and therefore will aid in the fundamental understanding, as well as the risk assessment, of lung fibrosis caused by exposure to CNTs, other nanomaterials, and other fibrogenic particles and fibers. The knowledge obtained will also facilitate the identification of new drug targets and biomarkers for the treatment and exposure and disease monitoring of human lung fibrosing diseases.

## Carbon nanotube-induced lung fibrosis

**Fig. 1:**
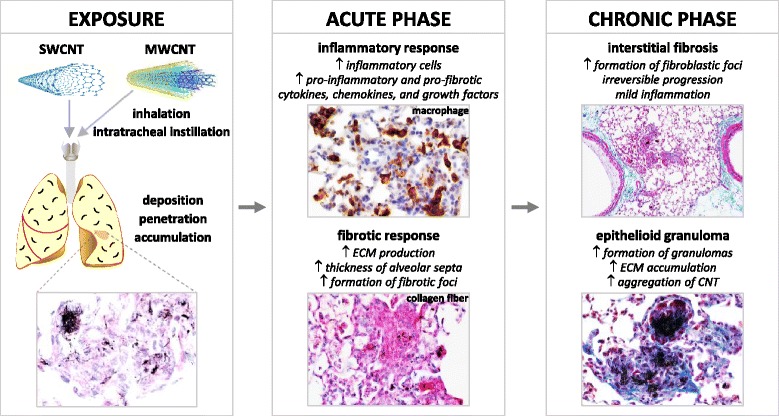
CNT-induced lung fibrosis. CNTs are respirable fibers with a tendency to deposit, penetrate and accumulate in lung tissues (*left box*). Exposure to CNTs induces acute phase responses including an inflammatory response, represented by the recruitment of Mac2-positive macrophages, and a fibrotic response, shown by Picro-Sirius Red staining for collagens I and III. Acute phase responses start as early as day 1, reach an apex on day 7, and decline after day 7 to significantly lower levels on day 14 post-exposure. In this scenario, day 7 post-exposure may represent an acute-to-chronic transition of CNT-induced pathology in mouse lungs (*middle box*). CNT-induced chronic phase responses are characterized by interstitial fibrosis and formation of epithelioid granulomas, shown by Masson’s Trichrome staining for collagen fibers on day 28 post-exposure. CNT-induced lung fibrosis appears to be persistent and irreversible in studies for up to 1 year post-exposure (*right box*)

CNT-induced lung interstitial fibrosis initiates with a prominent acute inflammatory response, exhibited by recruitment and accumulation of inflammatory cells, including neutrophils, macrophages, and lymphocytes, and elevated secretion of pro-inflammatory and pro-fibrotic cytokines, chemokines, and growth factors, such as TNF-α, IL-1β, IL-6, MCP-1, TGF-β1 and PDGF-A (PDGF subunit A) [22, 24, 25, 43–47]. Along with the acute inflammation, MWCNTs trigger a rapid-onset fibrotic response, indicated by increased deposition of collagen fibers in alveolar septa, detectable as early as day 1 post-exposure [24]. The acute inflammatory and fibrotic responses reach an apex by day 7 post-exposure, after which the pathologic effects transit to chronic fibrosis. At the chronic phase, CNT-induced fibrotic lesions are featured with mild inflammation, thickened alveolar septa, increased deposition of ECM proteins, enhanced expression of fibrosis markers, and formation of fibrotic foci and epithelioid granulomas [27, 48]. Figure 1 depicts the pathologic features and transition of the acute and chronic lung fibrotic lesions induced by CNTs in rodents. The lung fibrotic response to CNT exposure resembles the pulmonary response to deposition of fibrogenic foreign bodies in the lungs. In particular, the pathologic features and dynamics of the pulmonary interstitial fibrosis induced by CNTs display a high similarity to those of pneumoconiosis and IPF, both of which are progressive, incurable, and poorly understood human fibrotic lung diseases [24, 28].

Notably, CNT-induced lung fibrosis appears to be persistent and irreversible, which differs from bleomycin-induced lung fibrosis. The fibrotic lesions induced by a single dose instillation of bleomycin generally resolve after 28 days post-exposure [12, 13], whereas CNT-induced lung fibrosis was prolonged and was observed 90 days after a single dose intratracheal instillation of SWCNTs in mice, shown by the significant formation of epithelioid granulomas [44]. Similarly, lung fibrosis was observed 90 days after a single dose intratracheal instillation of MWCNTs in rats, demonstrated by thickened alveolar wall and increased collagen deposition [49]. In a more recent study, the long-term effects of CNTs on lung fibrosis were specifically investigated. Either a single dose intratracheal instillation of SWCNTs or an inhalation exposure to SWCNT aerosol (5 h/day for 4 days) induced lung fibrosis in mice 1 year post-exposure, demonstrated by increased levels of collagens in the lungs and the presence of fibrotic histopathological phenotypes [50]. In a separate study with inhalation of MWCNT aerosol (5 h/day for 12 days, 4 times/week for 3 weeks), it was found that lung fibrosis in MWCNT-exposed mice, indicated by increased fibrous collagen in the alveolar region, displayed a progressive increase in the thickness of the alveolar septal connective tissue over time, i.e., 0.17 ± 0.02, 0.22 ± 0.02, 0.26 ± 0.03, 0.25 ± 0.02 and 0.29 ± 0.01 μm on days 1, 14, 84, 168 and 336 post-exposure, which was significantly higher than that of the clean-air control on days 84 and 336 post-exposure [27]. These observations reveal that CNTs induce progressive and persistent fibrosis in the lungs, which is not self-resolved even at 1 year post-exposure. From this prospect, studies on CNT-induced lung fibrosis are promising, as they would, at the very least, supplement the findings from the bleomycin model to better reflect human fibrotic lung diseases, such as IPF and pneumoconiosis.

Some CNTs are capable of inducing bronchial or pleural fibrosis. In the former case, inhalation of MWCNTs in ovalbumin-sensitized mice, a murine asthma model, induced significant airway fibrosis in addition to asthmatic phenotypes [25]. In the latter case, direct instillation of long or short MWCNTs into the pleural cavity induced fiber length-dependent fibrotic responses like asbestos fibers [26]. The long MWCNTs elicited acute inflammation followed by progressive fibrosis on the parietal pleura, whereas the short MWCNTs were rapidly cleared through the stomata of the pleura and as a result, failed to induce pleural fibrosis. In both bronchial and pleural fibrosis, the development of fibrotic lesions involves acute inflammation followed by fibrotic progression similarly to that of interstitial fibrosis described above. The CNT-induced airway and pleural fibrotic lesions may have implications for the study of human asthma and pleural thickening/mesothelioma, respectively, which requires further investigation.

CNTs differ considerably from one another in their physicochemical properties that may impact CNT toxicity in vivo by affecting both the intrinsic pathogenicity and the kinetic behavior of CNTs [18]. Cumulative evidence reveals that the size, shape, surface area, surface reactivity, fiber rigidity, and biopersistence of CNTs are among the important properties to influence their fibrogenic activity in the lungs. The knowledge obtained from these structure-activity relationship studies provides insights into the internal (effective) dose of CNTs in lung fibrosis and hence their risk assessment. The information would also suggest new ways of reducing CNT fibrogenic activity by means of prevention-through-product design.

CNTs exhibit length-dependent activities in causing lung interstitial fibrosis [51, 52], as well as the fibrosis and granuloma formation in the parietal mesothelium [26]. In the latter case, the length-fibrosis correlation can be explained by the observation that long, but not short, CNTs (i.e., >15 μm in length) are retained in the pleural cavity to cause the fibrotic lesions, because the long CNTs are not efficiently cleared off through either the stomatal drain (3–10 μm in diameter) or pleural macrophage phagocytosis (<10–15 μm in diameter), whereas the short CNTs are rapidly eliminated from the pleural cavity through both clearance mechanisms [17]. It is believed that the long CNTs that exceed the diameter of macrophages and therefore are not effectively engulfed by macrophages would trigger the so-called “frustrated phagocytosis”. In this scenario, macrophages that fail to phagocytize the fibers are activated to release an array of bioactive and/or cytotoxic agents, which would cause tissue damage locally, much like the response to long asbestos fibers [17]. It is also possible that CNTs interact with cell surface structures, such as the pattern recognition receptors, to elicit responses from lung cells. In this case, the long CNTs may have a higher capacity of stimulating the cell surface receptors than the short CNT fibers to account for the differential fibrotic effects between long and short CNTs. MWCNTs have been shown to bind to the cell surface bone morphogenetic protein receptor type II (BMPR2) to stimulate the differentiation and to inhibit the apoptosis of mouse myoblast cells [53]. However, evidence supporting a direct interaction of CNTs with cell surface receptors to stimulate lung fibrosis is currently lacking.

It is worth noting that many CNTs are shorter than 5–10 μm and thus would be engulfed by macrophages if not agglomerated or tangled into large masses. Yet these CNTs may still be fibrogenic both in vitro and in vivo. Moreover, the lung parenchyma does not appear to have a “sieve” mechanism like the pleural stomata. Therefore, the length-dependent activities of CNTs in causing lung interstitial fibrosis may differ from those defined in the “fiber length pathogenicity paradigm” derived from mesothelial fibrosis caused by asbestos fibers with regard to both their phenotypes and mechanisms. For instance, the needle-like MWCNTs, i.e., Mitsui XNRI MWNT-7, have a mean length of 3.86 μm and count mean diameter of 49 nm, but are potent in inducing lung fibrosis in vivo, suggesting that properties other than fiber length, in particular, the fiber rigidity in the case of XNRI MWNT-7, play an important role in modulating the fibrogenic activity of nanomaterials [23, 24, 27].

A recent comparison among ten commercial MWCNTs with different morphology, composition, surface area, and functionalization reveals that surface area, fiber length, and surface modifications were better predictors of pulmonary inflammation in vivo [54]. In other studies, the thin film coating of MWCNTs with Al_2_O_3_ via atomic layer deposition reduced lung fibrosis in mice [55]; and the surface functionalization of MWCNTs with carboxylation or other covalent modifications demonstrates that strong cationic modifications induced significant lung fibrosis, whereas carboxylation significantly reduced the extent of lung fibrosis, compared with pristine CNTs [56]. Therefore, the surface reactivity and surface charge of CNTs play critical roles in determining their fibrogenicity and toxicity.

## Role of myofibroblasts and fibroblasts in lung fibrosis

**Fig. 2:**
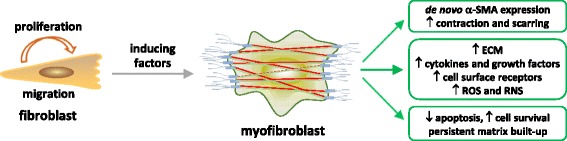
Fibroblasts and myofibroblasts in fibrosis. Fibroblasts and myofibroblasts act as the effector cells in organ fibrosis. Upon fibrogenic stimulation, tissue resident fibroblasts are activated to migrate and proliferate. Activated fibroblasts are the major progenitor cells to differentiate into myofibroblasts, indicated by the *de novo* expression of α-SMA. Myofibroblasts possess several characteristics, which distinguish them from fibroblasts and render them unique and critical functions in organ fibrosis

The fibroblastic response in lung fibrosis is responsible for the fibrotic matrix built-up, ECM remodeling, and tissue contraction to result in lung scarring and destruction. This dynamic process involves both myofibroblasts and fibroblasts that play distinct, but sometimes overlapping, functions; moreover, the interplay between these two types of cells represents an important aspect of fibrosis development, as illustrated in Fig. 2.

Resident fibroblasts are believed to be activated and play important roles in the early phase of wound healing and tissue fibrosis. Activated fibroblasts migrate, proliferate, and secrete certain ECM proteins, cytokines, and growth factors, to promote the fibrosis development at the site of injury. Among these functions, fibroblast proliferation has received increasing attention in fibrosis development. In this regard, the fibroblastic foci in IPF exhibit a prominent accumulation of proliferating fibroblastic cells that are fibroblasts in nature [37, 57]. Fibroblasts isolated from IPF patients show enhanced proliferation in vitro. Furthermore, a number of cellular mechanisms have been identified to account for the pro-proliferative state of these cells. There was an aberrant activation of PI3K/Akt signaling and pathological proliferation in these fibroblasts, which involved a decreased level of the plasma membrane integrin β1/caveolin-1/PTEN complex and a decreased activity of PTEN phosphatase [58]. Activated Akt phosphorylates and inactivates transcription factor FoxO3a (forkhead box O3a) that promotes cell cycle arrest by increasing the expression of CDK inhibitor p27. In a separate study, IPF fibroblasts were shown to have a high level of inactive FoxO3a due to a high Akt activity, which resulted in a reduced level of p27 and, consequently, increased proliferation of the cells [59]. A decreased level of integrin α2β1 and an impaired activity of PP2A phosphatase were also detected in IPF fibroblasts; these changes elevated the levels of inactive, phosphorylated GSK-3β and active nuclear β-catenin, both of which promote fibroblast proliferation [60]. In addition, the IPF fibroblasts display reduced apoptosis. The increased proliferation and reduced apoptosis of fibroblasts expand the fibroblast population and would therefore boost the fibroblast-to-myofibroblast transformation, which ultimately leads to the formation of fibroblastic foci and tissue fibrosis [61–63].

Myofibroblasts represent a heterogeneous group of fibroblastic cells found in tissues undergoing wound healing or organ fibrosis, and within the microenvironment of primary and metastatic cancers [34, 64]. As shown in Fig. 2, myofibroblasts exhibit several notable and unique characteristics that are distinct from those of fibroblasts and are believed to be critical to wound healing, fibrosis, tumorigenesis and cancer metastasis [34, 35, 65–67]. First, myofibroblasts possess a high contractile activity through their *de novo* synthesis of α-SMA protein. Newly synthesized α-SMA incorporates into intracellular stress fibers that are capable of generating strong force upon contraction, through which myofibroblasts control the cell shape and movement, ECM reorganization, and tissue contraction. Second, myofibroblasts possess a high capacity of protein synthesis and secretion and are believed to be responsible for the production of a major portion of the ECM proteins, such as collagens and fibronectin, for matrix deposition during fibrosis [38, 68–70]. Third, myofibroblasts demonstrate high levels of constitutive and induced expression of cytokines, chemokines, growth factors, and cell surface receptors, which render the cells some properties of inflammatory cells to allow them to respond to a variety of inflammatory, immune, and mechanical signals [71–75]. Fourth, myofibroblasts produce and release reactive oxygen and nitrogen species (ROS, RNS) spontaneously and under stimulation, which contribute to the up-regulation of ECM production and remodeling [76–80]. Last but not least, myofibroblasts in fibrotic tissues show high resistance to apoptosis, possibly due to the persistent activity of TGF-β1 and ECM deposition, leading to a prolonged survival and activity of myofibroblasts during the development of fibrosis [61, 81–83]. Combined, these features of myofibroblasts enable the cells to perform unique, direct, and critical functions in a broad range of physiologic and pathologic processes involving ECM remodeling.

The formation of myofibroblasts involves a complex and as yet not well understood process. In both IPF and bleomycin-induced lung fibrosis, activated interstitial resident fibroblasts are recognized as a major source of myofibroblasts. Myofibroblasts may also derive through trans-differentiation from other types of cells, such as the bone marrow-derived fibrocytes, the pericytes surrounding small blood vessel walls, and the epithelial cells overlying connective tissues. However, the contribution of these trans-differentiated cells to the myofibroblast pool in lung fibrosis remains a subject of debate, as studies examining the role of the trans-differentiated cells in fibrosis development have yielded inconsistent results in different experimental systems [41, 66, 84–88].

The differentiation of myofibroblasts from fibroblasts can be induced by a variety of signals. The transforming growth factor-β (TGF-β; mainly TGF-β1) has been recognized as a central player in driving fibroblasts to differentiate into myofibroblasts in both experimental and clinical settings, though a panel of other factors including ED-A fibronectin, Wnt, NOX4, integrins, ROS, and the stiff ECM have also been shown to promote myofibroblast differentiation, as have been summarized by Hinz and colleagues [41]. TGF-β1 stimulates all characteristics of the differentiated myofibroblasts both in vitro and in vivo [34]. Exaggerated expression and activation of TGF-β are commonly observed in lung fibrotic lesions, such as IPF and bleomycin-induced lung fibrosis [89–93]. Newly synthesized TGF-β is secreted into the ECM in the form of a latent complex [94]. The mechanisms by which TGF-β is induced and activated during fibrosis are not well understood and remain to be a topic of intensive research, which will be discussed in more detail in the section on mediators and mechanisms for CNT-induced lung fibrosis.

## Myofibroblasts in carbon nanotube-induced lung fibrosis

Given the critical role of myofibroblasts in lung fibrosis and the predominant pathologic phenotypes of lung fibrosis induced by CNTs described above, the potential effects of CNTs on myofibroblast differentiation and function have drawn attention in recent years.

In vitro studies have provided evidence demonstrating that CNTs stimulate the formation of myofibroblasts from fibroblasts and other types of cells. Treatment of macrophages (RAW264.7) with MWCNTs (average length 10 μm, average outer diameter 8.7 nm, surface area 220 m^2^/g, total metal 0.2 %) stimulated the cells to produce a range of pro-fibrotic cytokines and growth factors including TNF-α, IL-1β, TGF-β1, and PDGF at both mRNA and protein levels [95]. Induction of the proteins correlated with the activation of the NF-κB signaling pathway. Moreover, the cell-free medium from the treated culture was found capable of stimulating fibroblasts (WI38-VA13) to differentiate into myofibroblasts, as evidenced by significantly elevated expression of α-SMA protein. Similarly, SWCNTs (fiber length 0.5–2 μm, outer diameter 1–2 nm, surface area 480 m^2^/g, total metal 4.5 %) activated NF-κB and induced the expression of TGF-β1 in macrophages to produce a medium that induced the formation of myofibroblasts from lung fibroblasts [96]. These findings suggest a molecular mechanism by which CNTs stimulate a coordinated response between macrophages and fibroblasts to induce the differentiation of fibroblasts into myofibroblasts via secreted soluble factors, such as TGF-β1 and IL-1β.

CNTs induce the epithelial-mesenchymal transition (EMT), i.e., the trans-differentiation of epithelial cells to mesenchymal cells, to contribute to myofibroblast formation. During this process, epithelial cells gradually acquire a mesenchymal (fibroblast-like) phenotype through the *de novo* expression of vimentin, FSP-1 (fibroblast specific protein-1), Collagen I, fibronectin, and α-SMA. In a culture of human alveolar epithelial cells (A549), long MWCNTs (length: 5–15 μm) were shown to down-regulate the epithelial cell marker E-cadherin and up-regulate the expression of α-SMA protein, both of which indicate EMT [97]. MWCNTs also induced EMT in rat alveolar type II epithelial cells (RLE-6TN), shown by decreased E-cadherin expression and increased fibronectin expression [98]. These findings reveal that myofibroblasts can derive from epithelial cells through EMT when exposed to CNTs in vitro.

The involvement of myofibroblasts in CNT-induced lung fibrosis in vivo was addressed in a few recent studies. CNTs stimulate the expression of α-SMA mRNA and protein in the lungs of rodents. One study was focused on the effect of SWCNTs on EMT in the lungs wherein the expression of α-SMA and surfactant protein C (SPC, a marker of alveolar epithelial cells) was used as markers to trace cell differentiation and trans-differentiation [99]. Immunofluorescence staining showed that the α-SMA expression occurred in a portion of hyperplastic SPC positive epithelial cells in the lungs on days 28 and 42 post-exposure to SWCNTs; whereas, flow cytometry analysis revealed that about 17, 30, 33, and 29 % of the α-SMA positive cells were SPC positive on days 14, 28, 42 and 56 post-exposure to SWCNTs, respectively. These data demonstrate that a subset of α-SMA positive cells were differentiated from the epithelium-derived fibroblasts under SWCNT exposure. Although the overall effect of SWCNTs on α-SMA expression and myofibroblast differentiation in the lungs was not assessed, this study was the first to report α-SMA induction in CNT-exposed lungs. In a separate study, the toxicity of functionalized SWCNTs in mouse lungs was analyzed [100]. The protein level of α-SMA was significantly increased in lung tissues on day 14 post-exposure to certain surface-modified and functionalized SWCNTs. Therefore, SWCNTs are capable of inducing myofibroblast transformation in the lungs, as shown by increased number of α-SMA positive cells in lung tissues. In another report, the induction of EMT by short (length: 350–700 nm) and long (length: 5–15 μm) MWCNTs was investigated in mouse lungs with α-SMA as a marker of the mesenchymal cells [97]. The percentage of SPC positive cells stained for α-SMA in the lungs was significantly elevated by long, but not short, MWCNTs on days 28 and 56 post-exposure. Thus, MWCNTs induce α-SMA expression in SPC positive cells in the lungs through EMT in a fiber length-dependent manner.

**Fig. 3:**
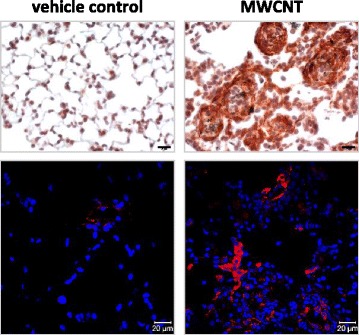
α-SMA expression induced by MWCNTs in mouse lungs. Pulmonary exposure to MWCNTs (XNRI MWNT-7, 40 μg) for 7 days strongly induces α-SMA expression. α-SMA expression in well-formed fibrotic foci is shown by immunohistochemistry (*upper panel*) and in less well-formed, early stage fibrotic foci by immunofluorescence (*lower panel*), respectively (scale bar: 20 µm; for immunofluorescence,* red*: α-SMA staining, *blue*: nuclear DAPI staining)

In an attempt to assess the effect of CNTs on myofibroblast transformation during lung fibrosis directly, we conducted a study in mouse lungs exposed to MWCNTs (XNRI MWNT-7, median length 3.86 μm, mean diameter 49 nm, average surface area 26 m^2^/g, total metal 0.78 %), a potent fibrogenic agent that induces rapid-onset lung fibrosis in mice [23, 24, 43]. We found that a single dose pharyngeal aspiration of MWCNTs (40 μg) significantly induced the expression of α-SMA protein and increased the number of α-SMA positive cells in mouse lungs on days 1, 3, 7 and 14 post-exposure. The increases were especially apparent in the interstitial fibrotic foci where MWCNTs deposited, as revealed by immunohistochemistry and immunofluorescence^1^. Representative images showing the induced expression of α-SMA on day 7 post-exposure are presented in Fig. 3, which demonstrate, for the first time, that MWCNTs, exemplified by XNRI MWNT-7, can remarkably stimulate myofibroblast transformation for the formation of fibrotic foci during lung fibrosis in vivo.

Several ECM proteins other than α-SMA typically expressed and produced by myofibroblasts during organ fibrosis display increased expression in CNT-exposed lungs, indicating that the myofibroblasts under CNT exposure are active and functional with regard to fibrosis development. A number of studies using traditional methods of collagen analysis demonstrate remarkably increased amounts of collagen fibers in CNT-exposed lungs, exemplified by the investigations listed in Table 1 with references included therein. Elevated expression and deposition of ECM proteins in the interstitial tissues of CNT-exposed lungs, especially the fibrotic foci where CNTs deposited, were also observed by molecular biology techniques. In one study, long MWCNTs (length: 20–50 μm), but not short MWCNTs (length: 0.5–2 μm), induced the expression of Collagen I mRNA on day 30 and Collagen III mRNA on day 7 and day 30 post-exposure in rat lungs [52]. Certain forms of functionalized SWCNTs significantly increased the levels of Collagen I and Collagen III in mouse lung tissues on day 14 post-exposure [100]. We recently demonstrated that MWCNTs (XNRI MWNT-7, 40 μg) caused significantly elevated mRNA expression of Col1a1 and Col1a2, the genes encoding the pro-alpha1 and pro-alpha2 chains of Collagen I, on days 3 and 7 post-exposure in mouse lungs [24, 101]. The treatment also dramatically increased the production and accumulation of Collagen I in interstitial lung tissues on days 1, 3, 7 and 14 post-exposure. A dose-dependence study showed that the induction of Collagen I accumulation by MWCNTs occurred at low doses, i.e., 5 and 20 μg, on day 7 post-exposure [101]. Additionally, it was detected that the expression of fibronectin mRNA was significantly increased on day 3 and day 7, and the level of fibronectin protein was markedly elevated on day 7, in mouse lungs exposed to MWCNTs (XNRI MWNT-7, 40 μg) [24].

**Table 1:** Increased pulmonary collagen deposition by CNTs as revealed by traditional assays

CNT	Length	Animal	Exposure	Method	Reference
SWCNT inhalation	0.1–1 μm	mouse	1–28 days	Sircol soluble collagen assay	[175]
				Picro-Sirius Red staining	
	1–3 μm	mouse	1 y	Sircol soluble collagen assay	[50]
SWCNT intratracheal injection	0.5–1.5 μm	mouse	14 days	Masson’s Trichrome staining	[100]
SWCNT pharyngeal aspiration	1–3 μm	mouse	7 days	Sircol soluble collagen assay	[176]
	5–15 μm	mouse	14–56 days	Hydroxylproline assay	[99]
				Masson’s Trichrome staining	
	1–3 μm	mouse	1 y	Sircol soluble collagen assay	[50]
MWCNT inhalation	3.86 μm	mouse	2–12 days	Masson’s Trichrome staining	[43]
	0.3–50 μm	mouse	1–98 days	Masson’s Trichrome staining	[47]
	4.3 μm	mouse	1–336 days	Picro-Sirius Red staining	[27]
	5.5–6.2 μm	rat	90 days	Masson’s Trichrome staining	[46]
MWCNT intratracheal injection	20–50 μm	rat	1–30 days	Picro-Sirius Red staining	[52]
	0.7 μm	rat	60 days	Hydroxylproline assay	[45]
	5.9 μm			Masson’s Trichrome staining	
MWCNT pharyngeal aspiration	3.86 μm	mouse	1–14 days	Masson’s Trichrome staining	[24]
				Picro-Sirius Red staining	
	0.5–40 μm	mouse	28 days	Masson’s Trichrome staining	[55]
	3.86 μm	mouse	1–56 days	Picro-Sirius Red staining	[23]

Induction of the platelet-derived growth factor receptor-β (PDGFR-β) expression is another feature of myofibroblast activation during fibrosis in several organs including the liver, kidney and lung [102–105]. By using a double-fluorescent *Pdgfrb*-Cre reporter mouse strain, it was demonstrated that, in the lungs following bleomycin treatment for 28 days, a marked expansion of the reporter cells occurred in fibrotic regions, in which almost all the reporter cells expressed PDGFR-β and most of the cells expressed α-SMA, indicating that a high percentage of PDGFR-β-expressing cells are lung myofibroblasts [105]. Therefore, an induced expression of PDGFR-β is a marker for myofibroblast activation during fibrogenesis. We examined the level of PDGFR-β in MWCNT-induced lung fibrosis and demonstrated that MWCNTs (XNRI MWNT-7, 40 μg) remarkably increased the PDGFR-β expression and the number of PDGFR-β positive cells in mouse lungs on day 7 post-exposure, especially in interstitial fibrotic foci^1^. These findings further support the activation of myofibroblasts by CNTs in the lungs in vivo.

## Candidate mediators and mechanisms for CNT-induced myofibroblast differentiation and function

A number of signaling molecules and cellular processes induced by CNTs have been suggested to play important roles in myofibroblast differentiation and activation. In this section, we discuss evidence supporting their involvement and their potential mechanisms in the regulation of myofibroblast formation and behaviors in CNT-induced lung fibrosis with the goal to gain insights into the molecular underpinnings of induced lung fibrosis.

## TGF-β1

**Fig. 4:**
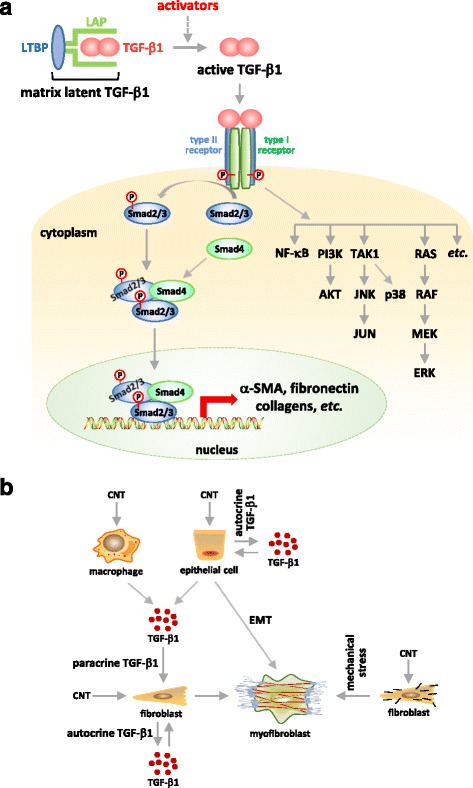
Regulation of myofibroblast formation by TGF-β1. **a** Schematic presentation of TGF-β1 signaling in myofibroblast formation. Upon stimulation, latent TGF-β1 is activated and active TGF-β1 is released to bind to its receptors on the cell surface to drive the Smad-dependent pathway, which directly up-regulates the transcription of fibrotic genes encoding α-SMA, collagens, and fibronectin. Binding of active TGF-β1 to its receptors also elicits a number of Smad-independent pathways, such as the PI3K-AKT signaling, which may promote myofibroblast differentiation and function. **b** Role of TGF-β1 in CNT-stimulated myofibroblast differentiation. CNTs induce the production and secretion of TGF-β1 by macrophages and epithelial cells, which serves as a paracrine factor to stimulate fibroblast-to-myofibroblast differentiation. CNTs also directly induce fibroblasts to produce and secrete TGF-β1, which functions as an autocrine factor for fibroblasts to differentiate into myofibroblasts. CNTs may directly promote fibroblast-to-myofibroblast differentiation by mimicking the ECM or intracellular collagen fibers to generate mechanical stress. In addition, CNTs stimulate epithelial cells to produce and secrete TGF-β1, which may induce the trans-differentiation of epithelial cells to myofibroblasts via EMT

TGF is a superfamily of more than sixty structurally related growth factors that regulate many different physiological and disease processes across species. TGF-β1, along with its isoforms TGF-β2 and 3, is a prototype of the TGF superfamily. TGF-β1 inhibits the proliferation of most types of cells and induces the apoptosis of epithelial cells; conversely, it stimulates mesenchymal cells to proliferate and differentiate into myofibroblasts, which prompts wound healing or tissue fibrosis in various organs [106]. Evidence supports the notion that TGF-β1 is arguably the most predominant pro-fibrotic growth factor in vivo. For instance, TGF-β1 expression is elevated in lung fibrotic lesions, such as IPF and bleomycin-induced lung fibrosis [90–92]; TGF-β1 induces fibroblast activation in vitro and overexpression of active TGF-β1 leads to persisting lung fibrosis in vivo [107, 108]; knockout of the TGF-β1 gene in mice causes severely impaired wound repair alongside severe wasting, generalized inflammation, and tissue necrosis leading to organ failure and death, whereas the epithelium-specific deletion of TGF-β receptor type II protects mice from bleomycin-induced lung fibrosis [109, 110]; lastly, blocking TGF-β1 signaling ameliorates lung fibrosis in animal models [111, 112]. Relevant to this review, TGF-β1 has been recognized as one of the most important endogenous regulator to drive myofibroblast differentiation and activation, because it directly controls the *de novo* expression of α-SMA and the induced expression of ECM proteins including collagens and fibronectin; moreover many fibrogenic signals activate myofibroblast functions by modulating the expression and/or activation of TGF-β1, or by cross-interacting with TGF-β1 signaling in and outside of the cell (Fig. 4a) [34, 41].

As a pro-fibrotic growth factor, TGF-β1 can be induced by injury and other fibrosis-stimulating signals from several types of cells including bone marrow-derived cell lineages, such as macrophages, neutrophils, and T lymphocytes, as well as structural cells, such as airway epithelial cells, endothelial cells, and mesenchymal cells, i.e., fibroblasts and myofibroblasts [34, 90, 113–115]. Newly synthesized TGF-β1 is confined within the latency-associated peptide (LAP) and is further associated with the latent TGF-β binding protein (LTBP). This large latency complex (LLC) is secreted into the ECM where it anchors to the ECM by binding to fibronectin and Fibrillin 1 through LTBP. This configuration keeps TGF-β1 in a latent state that needs to be released from the complex to be activated. Indeed, cumulative evidence reveals that, in addition to induced expression of TGF-β1 mRNA and protein, much of the regulation of TGF-β1 in physiologic and pathologic processes centers on the activation of the latent TGF-β1 [92, 94]. Upon activation, TGF-β1 binds to its receptors to form a complex consisting of a TGF-β1 homodimer, two TGF-β type I receptors, and two TGF-β type II receptors on the cell surface. The formation of the TGF-β1-receptor complex leads to the activation of the Smad-dependent transcription of fibrotic genes encoding α-SMA, collagens, fibronectin, *etc.*, as well as Smad-independent signaling, to drive myofibroblast differentiation from fibroblasts (Fig. 4a).

CNTs have been shown to increase the level of TGF-β1 protein in vitro and in vivo in several types of cells (Fig. 4b). Treatment of mouse leukemic monocyte-macrophage RAW264.7 cells with either MWCNTs or SWCNTs significantly induced the expression of TGF-β1 mRNA and protein; moreover, the cell-free and CNT-free supernatant of a conditioned medium from the treatment stimulated human normal lung fibroblasts (WI38-VA13) to differentiate into myofibroblasts, as indicated by the induced expression of α-SMA [95, 96]. In separate studies, RAW264.7 macrophages exposed to SWCNTs displayed a potent induction of secreted TGF-β1 in the culture medium [22], and RAW264.7 macrophages exposed to long MWCNTs (length: 20–50 μm) had a remarkably increased expression of TGF-β1 mRNA as well as an elevated level of secreted TGF-β1 in the culture medium [52]. Induced expression of TGF-β1 mRNA and protein was also observed in human normal bronchial epithelial cells (BEAS-2B) treated with either MWCNTs or SWCNTs; and the level of secreted TGF-β1 in the culture medium of BEAS-2B cells was increased by SWCNTs [95, 96, 116]. In the above cases, TGF-β1 produced from macrophages and epithelial cells stimulates the differentiation of fibroblasts to myofibroblasts as a paracrine factor.

CNTs also induce the production and secretion of TGF-β1 from fibroblasts directly. SWCNTs induced TGF-β1 expression and promoted rat vascular adventitial fibroblasts to transform to myofibroblasts, indicated by the gained expression of SM22-α (smooth muscle protein 22-α), which is a smooth muscle cell-specific protein and a marker of myofibroblast differentiation [117]. SWCNTs induced TGF-β1 secretion and activation in a dose-dependent manner in human lung fibroblasts (CRL-1490) [118]. Long and short SWCNTs with median lengths of 12.31 and 1.13 μm, respectively, induced TGF-β1 expression and secretion in normal human lung fibroblasts (NHLF); but the induction by the long SWCNTs was significantly more pronounced than that by the short ones [119]. SWCNTs and MWCNTs increased the protein expression of TGF-β1 and Collagen I in CRL-1490 cells as well as the level of secreted TGF-β1 in the culture medium [120]. MWCNTs also induced the expression of α-SMA mRNA and protein in mouse embryonic fibroblasts (NIH 3T3) [98]. In the above cases, CNT exposure activated fibroblasts to produce TGF-β1, which functions as an autocrine factor to induce α-SMA expression and promote the fibroblast-to-myofibroblast differentiation.

Elevation of the TGF-β1 protein level in the bronchoalveolar lavage (BAL) fluid provides a measurement of TGF-β1 induction in vivo. SWCNTs remarkably increased the level of TGF-β1 in the BAL on day 7 post-exposure in mice [22]. SWCNTs and MWCNTs elevated the level of TGF-β1 in the BAL on day 21 post-exposure in mice [56, 116, 121, 122]. Long MWCNTs (length: 20–50 μm) induced an elevated level of TGF-β1 in the BAL on day 1 and in alveolar macrophages on day 7, as well as an increased level of Smad2 phosphorylation, a marker of the activation of TGF-β1 signaling, on day 7 post-exposure in rat lungs [52]. These findings implicate TGF-β1 signaling in CNT-induced lung fibrogenesis. In a separate study, long MWCNTs (length: 5–15 μm), but not short MWCNTs (length: 350–700 nm), increased the level of TGF-β1 in the BAL on days 7 and 28 post-exposure; moreover, the percentage of SPC and α-SMA double positive cells in mouse lungs was significantly elevated by the long MWCNTs, but not the short MWCNTs, on days 28 and 56 post-exposure, suggesting that the induction of TGF-β1 by MWCNTs leads to α-SMA expression in SPC positive cells in the lungs, possibly through the trans-differentiation of alveolar epithelial cells to myofibroblasts [97].

The widely used and well characterized MWCNTs in the study of CNT-induced lung fibrosis, XNRI MWNT-7, are intermediate in length (median length: 3.86 μm) compared with the long and short MWCNTs discussed above. MWNT-7 CNTs have been shown to significantly increase the levels of TGF-β1 protein in mouse BAL on days 3, 7 and 14 post-exposure, and in mouse lung tissues, especially in the regions where fibrosis occurs, on day 7 post-exposure [24]. These findings demonstrate a remarkable induction of TGF-β1 as an acute response to MWCNTs, which might control myofibroblast differentiation and activation, indicated by highly induced expression of α-SMA, Collagen I, and fibronectin during the acute phase response, in MWCNT-exposed lungs [24]^1^. Taken together, these studies support that TGF-β1 is significantly induced and may play a critical role in promoting myofibroblast differentiation and activation in CNT-triggered lung fibrosis.

A number of mechanisms have been described to account for the activation of the latent TGF-β1 stored in LLC, some of which have implications for CNT-induced lung fibrosis. Activation of the latent TGF-β1 can occur (a) by way of cellular acidification that denatures LAP to release TGF-β1, (b) via ROS that oxidize LAP to perturb the interaction between LAP and TGF-β1, (c) through thrombospondin-1 (TSP-1) that directly interacts with the latent complex to prevent it from binding matured TGF-β1, (d) by proteases including plasmin, MMP-2 and MMP-9, tryptase, elastase, and thrombin that activate TGF-β1 by proteolytic degradation of LLC and then LAP of the latent TGF-β1 complex, and (e) through integrins, such as the epithelial cell-specific αvβ6, which mediates matrix contraction to release active TGF-β1 close to the cell surface, and the fibroblast αvβ8, which presents latent TGF-β1 to a membrane-bound protease (i.e., MT1-MMP) to activate TGF-β1 [123, 124]. It is known that both MWCNTs and SWCNTs stimulate the production of ROS in vitro and in the lungs [95, 96, 101, 125], which would induce the activation of TGF-β1 in a manner analogous to that of asbestos [126, 127]. In recent studies, we have shown that the lung expression of TSP-1 was significantly induced by MWCNTs in vivo [101], whereas that of MMP-2 was induced during lung fibrosis from exposure to silica, paraquat, or bleomycin [28]. Direct evidence supporting a role of these mediators in the activation of latent TGF-β1 in CNT-induced lung fibrosis awaits further investigation.

Certain SWCNTs and MWCNTs have been shown to induce EMT to generate myofibroblasts in the lungs, which is accompanied by the activation of TGF-β1 signaling [97, 99]. These studies suggest that TGF-β1 may induce myofibroblast formation through EMT in CNT-exposed lungs. However, studies on the contribution of myofibroblasts derived via EMT in IPF and in bleomycin-induced lung fibrosis, as well as in fibrosis occurring in other organs such as kidney and liver, are controversial; thus, the role of EMT in fibrosis remains uncertain currently [88, 128]. Evaluation on whether and, if so, how much the TGF-β1-regulated myofibroblast trans-differentiation through EMT contribute to CNT-induced lung fibrosis might provide an answer to this question with regard to the phenotype, mechanism, and function of EMT.

## PDGF

The platelet-derived growth factor (PDGF) represents another important pro-fibrotic growth factor that confers multiple functions in organ fibrosis including human and experimental lung fibrosis [128–130]. For instance, overexpression of PDGF-B (PDGF subunit B) in mouse lungs induced severe fibrosis [131]. Instillation of rats with bleomycin caused elevated levels of PDGF-AA (homodimer of PDGF subunit A) and PDGF-BB (homodimer of PDGF subunit B) in the BAL fluid; moreover, the concentrated BAL showed a growth-promoting activity toward lung fibroblasts that can be partially blocked with anti-PDGF-BB (64 %) or anti-PDGF-AA (15 %) antibodies [132]. PDGF is a potent mitogen for cells of a mesenchymal origin, such as fibroblasts, both in vitro and in vivo; and it boosts the recruitment and proliferation of fibroblasts, promotes the differentiation of myofibroblasts from fibroblasts and other types of cells, and increases the production of ECM proteins from myofibroblasts during the fibrosis of various organs [104, 128, 129, 133–135].

PDGF has been shown to be induced by CNTs in the lungs in a number of recent studies. PDGF-AA was significantly increased in mouse BAL on day 21 post-exposure to MWCNTs in a dose-dependent manner [56, 121]. During the early phase response to MWCNTs, PDGF-AA was shown to be significantly increased in the BAL on days 1, 3 and 7, and in lung tissues on day 7, post-exposure to XNRI MWNT-7 by aspiration in mice [24]. In a separate study, PDGF-AA was induced in the BAL on day 1 post-inhalation exposure to MWCNTs in mice [25]. Also, a study in rats revealed that PDGF-AA was induced in the BAL on day 1, and in lung tissues on day 1 and day 21, post-exposure to MWCNTs, which was boosted by co-stimulation with bacterial lipopolysaccharides [136]. These findings suggest the possibility for PDGF to play a role in promoting myofibroblast differentiation and activation in the lungs exposed to CNTs. However, further detailed studies are needed to ascertain this posit.

## Th2 cytokines IL-4 and IL-13

The T helper 2 (Th2)-type cytokines IL-4 and IL-13 have been studied intensively in a variety of fibrotic diseases and animal models, and have been demonstrated to function as potent pro-fibrotic mediators to drive fibrosis development [8, 128, 137, 138]. For instance, increased levels of IL-4 were detected in patients with IPF or cryptogenic fibrosing alveolitis [139, 140]; and inhibition of IL-4 by neutralizing antibodies or inhibitors reduced liver fibrosis and dermal fibrosis in mice [141, 142]. IL-13 levels were significantly higher in IPF patients than in normal controls [143]; overexpression of IL-13 in mouse lungs induced subepithelial airway fibrosis [144]; and inhibition of IL-13 by neutralizing antibodies decreased collagen deposition in mouse lungs exposed to bleomycin [145].

IL-4 and IL-13 receptors are located on the cell surface of a number of mouse and human fibroblast subpopulations [146, 147]. In multiple in vitro studies, it was shown that, under the stimulation of IL-4 or IL-13, fibroblasts displayed enhanced proliferation and differentiation, and increased production of α-SMA and ECM proteins, such as type I and type III collagens and fibronectin [146–152], which indicates that IL-4 and IL-13 signaling may promote fibrosis by stimulating fibroblast-to-myofibroblast differentiation and by enhancing tissue remodeling. The Th2-type response may also promote myofibroblast differentiation by activating TGF-β1. It has been reported that IL-13 activates TGF-β1 in two ways: first, IL-13 induces the production of latent TGF-β1 from macrophages [153]; second, IL-13 activates TGF-β1 by increasing the expression of proteins that function in the cleavage of LAP, which keeps TGF-β1 as an inactive form, such as matrix metalloproteinases (MMPs) and cathepsins [153–155]. Taken together, cumulative evidence reveals that IL-4 and IL-13 play critical roles in the initiation and development of fibrosis, which are in part mediated by inducing myofibroblast differentiation.

We recently demonstrated that IL-4 and IL-13 expression and signaling were significantly induced by MWCNTs (XNRI MWNT-7) in mouse lungs [156]. In a genome-wide microarray gene expression study of mouse lung tissues, Th2-driven immune responses were preferentially enriched. In particular, the activation of IL-4 and IL-13 signaling, the center of Th2-type responses, was a dominant effect induced by MWCNTs on day 7 post-exposure. Time-course studies detected that IL-4 was significantly induced by MWCNTs at the mRNA level on days 1, 3, 7 and 14, and at the protein level on days 3, 7 and 14, post-exposure. IL-13 was significantly induced at the mRNA and protein levels on days 3, 7 and 14 post-exposure. In addition, a panel of signature downstream target genes of IL-4/IL-13 signaling, such as Il4i1, Chia, and Ccl11/Eotaxin, were remarkably induced by MWCNTs at both the mRNA and protein levels, further supporting the activation of IL-4/IL-13 signaling. The increased expression of IL-4 and IL-13 during the early phase fibrotic response, i.e., days 1 to 14 post-exposure, strongly suggests the potential for IL-4 and/or IL-13 to play a role in promoting myofibroblast differentiation in MWCNT-exposed lungs to drive fibrosis development.

## ROS

Oxidative stress reflects a cellular stress state that occurs when the production of ROS and antioxidant defense are out of balance, which causes multiple damages to the cell, such as DNA strand breaks and DNA mutation, protein peptide chain breaks, and lipid peroxidation, leading to cell death in the severe case [157–159]. ROS have been implicated in promoting fibrosis in multiple organs, such as the lung, liver, and kidney, through a number of mechanisms, as have been discussed in several recent reviews [160–167]. Significantly, it has been established that ROS promote the transformation of fibroblasts to myofibroblasts by interacting with the TGF-β1 signaling pathway [168–172]. ROS can augment the expression and secretion of TGF-β1 and activate the latent TGF-β1 to become active and functional. In a reciprocal manner, TGF-β1 increases ROS production, mainly through the induction of NOX4 expression. The NOX4-dependent production of hydrogen peroxide (H_2_O_2_) is essential for TGF-β1-mediated myofibroblast differentiation and ECM production. These findings clearly demonstrate a necessary role of ROS in myofibroblast differentiation and activation.

In the studies on CNT-induced toxicity, numerous observations consistently demonstrate oxidative stress as a predominant mechanism to link CNT exposures to their toxicological and pathological outcomes. A large number of in vitro cell culture studies support that both SWCNTs and MWCNTs directly stimulate ROS production in various types of cells, such as macrophages, fibroblasts, and bronchial and alveolar epithelial cells, as summarized in two recent reviews [18, 173]. For instance, MWCNTs were shown to stimulate the production of ROS in macrophages to activate NF-κB signaling [95], whereas SWCNTs were found to stimulate both fibroblast proliferation and angiogenesis via the induction of ROS production [118]. Importantly, a few in vivo studies have confirmed that CNT exposure results in oxidative stress in tissues. Exposure of mice to SWCNTs led to a dose-dependent accumulation of 4-hydroxy-2-nonenal (4-HNE, a lipid peroxidation biomarker) in the BAL as early as 1 day post-exposure, and a dose- and time-dependent depletion of glutathione (GSH, a major antioxidant) in the lungs, demonstrating the presence of oxidative stress upon exposure to SWCNTs [22]. Another indicator of oxidative stress, heme oxygenase 1 (HO-1), has also been shown to have an increased level in mouse lungs, aorta, and heart on day 7 post-exposure to SWCNTs [174]. In the NADPH oxidase knockout mice that lack the gp91^phox^ (Nox2) subunit of a NOX enzymatic complex and are deficient in ROS production, lung fibrosis induced by SWCNTs or MWCNTs was remarkably attenuated, compared with the wild-type control mice [122, 125, 175, 176].

We analyzed the roles of ROS in MWCNT (XNRI MWNT-7)-induced pathologic effects on the lungs, by using the nuclear factor erythroid 2-related factor 2 (Nrf2)-deficient mice, which have an elevated oxidative stress due to the lack of the defense against oxidative stress mediated by Nrf2 [177, 178]. Under exposure to MWCNTs, several markers indicative of oxidative stress, including ROS production in alveolar macrophages, the levels of DNA oxidation indicators 8-OHdG (8-hydroxy-2′-deoxyguanosine) and γH2AX (phospho-Histone H2A.X (Ser139)) and the level of 4-HNE in lung tissues, were remarkably increased in the lungs; and the increases were markedly more pronounced in Nrf2 knockout lungs than in wild-type lungs. There was also a remarkably higher level of MWCNT-induced lung fibrosis in Nrf2 knockout lungs than in wild-type lungs [101]. Taken together, these in vivo studies strongly support that CNTs induce ROS production in the lungs, and ROS play an important role in the initiation and progression of CNT-induced lung fibrosis, suggesting that CNT-induced ROS can serve as an enhancer to promote myofibroblast differentiation in the lungs.

## Role of inflammation and pro-inflammatory cytokines

The role of inflammation in organ fibrogenesis is complex [8]. Acute inflammation precedes and sometimes accompanies fibrosis in most, if not all, lung fibrosis induced by exposure to fibrogenic and cytotoxic agents including chemicals, microbes, and particles and fibers. On the other hand, fibrosis in the absence of apparent tissue injury may occur without a prominent inflammatory phenotype; moreover, anti-inflammation alone does not effectively prevent or block the development of fibrosis. It is believed that the role of inflammation in fibrosis development varies among fibrosing diseases; but once present, increased inflammatory infiltration and secretion create a milieu rich in pro-fibrotic growth factors, cytokines, and chemokines that foster the development of fibrosis, in part mediated by priming or promoting fibroblasts to differentiate into myofibroblasts. In this context, a panel of pro-inflammatory cytokines including TNF-α, IL-1α, IL-1β, and IL-6 have been shown to be pro-fibrotic factors in both mouse and human lung fibrosis models [18, 33].

TNF-α and IL-1β are among the earliest cytokines recognized as pro-fibrotic factors. Overexpression of TNF-α in mouse lungs resulted in spontaneous lung fibrosis [179]. TNF-α appears to play important roles in various fibrosis animal models, such as bleomycin- or silica-induced lung fibrosis and CCl_4_-induced liver fibrosis, as well as a number of human fibrotic diseases, such as IPF and asbestosis [180–183]. IL-1β and its receptors have been shown to promote fibrosis in different types of organ fibrosis, whereas inhibition of IL-1β signaling reduces the fibrosis development, illustrating a critical role of IL-1β signaling in organ fibrosis [184–191]. The IL-1α-deficient mice exhibited reduced collagen deposition in lung tissues in response to bleomycin treatment [192]. Enhanced IL-6 level was detected in the BAL of IPF patients [193], and IL-6 signaling was found to be key to driving fibrosis in a mouse model of acute peritoneal inflammation [194]. Moreover, a number of studies demonstrate that these cytokines can stimulate mesenchymal cells from multiple organs to express α-SMA [128, 195–199]. How these pro-inflammatory and pro-fibrotic cytokines induce myofibroblast differentiation and activation at the molecular level remains to be delineated.

Upon exposure to CNTs, the lungs elicit an acute inflammatory response as a well-characterized feature of CNT-induced lung toxicity. In many cases, acute inflammation precedes and accompanies CNT-induced lung fibrosis. A number of studies have detected increased expression and production of these pro-inflammatory cytokines in CNT-exposed lungs. The level of TNF-α in the BAL was significantly increased by SWCNTs on day 1 post-exposure in mice [22], by MWCNTs at 12 h or on days 1, 3 and 7 post-exposure in mice [24, 55, 200–202], and by MWCNTs on day 3 post-exposure in rats [45]. The level of IL-1α in the BAL was significantly increased by MWCNTs (XNRI MWNT-7) on days 1, 3, 7 and 14 post-exposure in mice [24]. The level of IL-1β in the BAL was significantly increased by SWCNTs at 40 h or on days 1, 3, 7 and 28 post-exposure in mice [22, 116], and by MWCNTs on days 1, 3 and 21 post-exposure in mice [24, 55, 122, 201]. The level of IL-6 in the BAL was significantly increased by MWCNTs at 12 h or on days 1, 3, 7, 14 and 28 post-exposure in mice [24, 55, 200, 202]. These pro-inflammatory cytokines with increased production from the acute innate immune response may contribute to the induction of myofibroblast differentiation in CNT-exposed lungs.

While induced expression of pro-inflammatory cytokines remains a major mechanism of up-regulation of their signaling, the activation of inflammasome processing of cytokines IL-1β and IL-18 has been increasingly recognized as a critical process for a variety of host responses and diseases including fibrosis [203–205]. Inflammasomes are large protein complexes in the cytoplasm that sense extracellular and intracellular signals to initiate innate immune responses to microbe exposure and tissue injury. Particulate and fibrous materials, such as silica, asbestos, cholesterol crystals, and CNTs, have been shown to activate inflammasomes, mainly the NLRP3 inflammasome, to mediate the proteolytic maturation of IL-1β and IL-18 [188, 205–207]. Activation of the NLRP3 inflammasome increases the formation of myofibroblasts in bleomycin-induced skin fibrosis [204]. Given the broad and critical roles of IL-1β, it is believed that inflammasome activation plays an important role in myofibroblast formation and function in CNT-induced lung fibrosis. However, direct evidence supporting this notion awaits further investigation.

## Role of proliferation

During lung fibrosis, fibroblasts undergo an elevated proliferation through multiple mechanisms, which would promote the formation of fibroblastic foci and the destruction of lung tissues. As fibroblasts are the major progenitor cells of myofibroblasts, it is rational to posit that an increase in the number of fibroblasts leads to a higher number of myofibroblasts and the accumulation of myofibroblasts in fibrotic foci.

The induction of fibroblast proliferation by CNTs has been observed in a number of in vitro studies. SWCNTs induced the proliferation of human lung fibroblasts (CRL-1490) in a dose- and time-dependent manner, which was mediated by the ROS-regulated activation of p38 MAPK (mitogen-activated protein kinase) and the induction of TGF-β1 and VEGF (vascular endothelial growth factor) [118, 208]. MWCNTs stimulated the proliferation of multiple types of fibroblasts in tissue culture in a dose- and physicochemical property-dependent manner [51, 209]. Furthermore, MWCNTs directly promoted the proliferation of mouse lung fibroblasts (MLg cells) primed with a low concentration of growth factor TGF-β1 or PDGF, by prolonging the phosphorylation of the extracellular signal-regulated kinase (Erk) 1/2 [210]. CNTs may stimulate fibroblast proliferation by inducing the secretion of soluble factors from epithelial cells, which is NLRP3 inflammasome-dependent, but TGF-β1-independent [211]. These studies demonstrate that certain types of CNTs are capable of stimulating fibroblast proliferation directly and thereby contribute to CNT-induced lung fibrosis.

The tissue inhibitor of metalloproteinase 1 (TIMP1) is highly induced and is secreted into the ECM from macrophages and mesenchymal cells during lung fibrosis. Using the Timp1-deficient mice, we demonstrated that TIMP1 plays a critical role in the development of MWCNT-induced lung fibrosis^1^. In the lungs of wild-type mice, MWCNTs (XNRI MWNT-7) remarkably increased the proliferation of fibroblasts, indicated by the expression of cell proliferation markers Ki-67 (marker of proliferation Ki-67) and PCNA (proliferating cell nuclear antigen). However, this induction was significantly attenuated in the lungs of Timp1-deficient mice. Accordingly, the MWCNT-induced fibrotic responses, including the formation of fibrotic foci, the differentiation of myofibroblasts, and the production and deposition of ECM proteins, such as Collagen I and fibronectin, were significantly higher in wild-type mice than in Timp1-deficient mice. These findings strongly suggest that MWCNTs stimulate fibroblast proliferation in the lungs and thereby promote myofibroblast differentiation from the enriched fibroblast pool to boost fibrosis. Mechanistic analysis demonstrated that MWCNT-induced fibroblast proliferation might be mediated by the formation of a TIMP1/CD63/integrin β1 complex on the surface of fibroblasts, which promotes the Erk1/2 phosphorylation and activation in fibroblasts in the lungs. These findings establish a direct mechanistic link among MWCNT exposure, fibroblast proliferation, myofibroblast differentiation, and lung fibrosis in vivo.

## Role of tissue stiffness, mechanical force, and receptors

In the model of wound healing, the formation of myofibroblasts generally becomes a major event at 1 week after tissue injury, which correlates with a significantly increased tissue tension or stiffness [67]. In fact, it has been shown that the threshold stiffness for the *de novo* expression of α-SMA in stress fibers ranges around 20,000 Pa, which is about 200 to 2000-fold higher than that of the early wound provisional ECM (i.e., 10–100 Pa) [212]. This increase in tissue stiffness and myofibroblast formation correlates with the increased activation of TGF-β1 protein within the tissue matrix. Mechanistic studies demonstrate that, indeed, the increased stiffness is necessary for the activation of the latent TGF-β1 and the release of activated TGF-β1 from the large latency complex in the vicinity of the site of wound healing or fibrosis [41]. In particular, the mechanical tension generated through the contraction of myofibroblasts is a well-recognized mechanism for the activation of the latent TGF-β1 in the matrix [213]. Based on these findings, it is believed that the CNT-induced tissue injury and fibrotic changes stimulate myofibroblast contraction to increase tissue stiffness, which in turn boosts the differentiation of fibroblasts into myofibroblasts by activating the latent TGF-β1 locally. This chain of events sets in motion a positive feedforward response among myofibroblast formation, contraction, tissue tension, and TGF-β1 activation to drive organ fibrosis. However, a direct measurement of tissue stiffness of the lungs exposed to CNTs in relation to fibrosis and myofibroblast activation is needed to prove this notion.

At the molecular level, CNTs may modulate cellular and ECM mechanical properties through several means. In addition to stimulating the tissue to release soluble factors, such as TGF-β1, discussed above, CNTs may induce the contraction and increase the cellular tension of cells by interfering with the intracellular contractile structures once inside the cell, as some CNT fibers resemble the cytoskeletal or contractile filaments in size and shape. Alternatively, CNT fibers may directly interact with cell surface receptors, such as the pattern recognition receptors. SWCNTs have been observed inside rat vascular adventitial fibroblasts after exposure for 24 and 48 h [117]. MWCNTs were found to be accumulated on the surface of NIH 3T3 fibroblasts 3 h post-exposure, and some MWCNTs entered the cell by way of endocytosis 24 h post-exposure [98]. Moreover, MWCNTs were shown to bind to BMPR2 on the surface of myoblasts to modulate their differentiation [53]. These findings raise the possibility of a direct effect of CNTs on fibroblasts and myofibroblasts by way of mechanical activation of the matrix latent TGF-β1 or biochemical activation of cell surface receptor-mediated intracellular signaling, to stimulate the activation and differentiation of fibroblasts into myofibroblasts. In both scenarios, the physicochemical properties of CNTs, such as the nano-scaled diameter, fiber length, surface area and reactivity, and biopersistence, would be critical parameters to influence their stimulatory activities. Although attractive, these possibilities remain to be proven by direct evidence from studies using multiple cellular, molecular, and biophysical means in the future.

## Conclusion

**Fig. 5:**
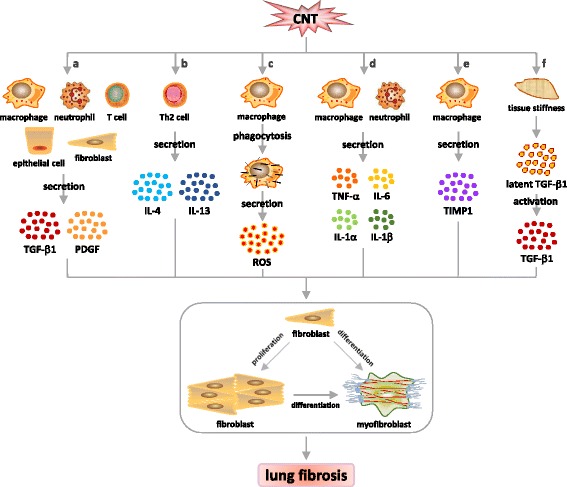
Mediators of CNT-induced myofibroblast differentiation in the lungs. CNTs stimulate multiple mechanisms and mediators capable of promoting myofibroblast formation and function, including **a** pro-fibrotic growth factors TGF-β1 and PDGF, **b** Th2 cytokines IL-4 and IL-13, **c** ROS, **d** pro-fibrotic cytokines TNF-α, IL-6, IL-1α and IL-1β, **e** TIMP1, and **f** tissue stiffness. Activation of these signaling cascades may induce myofibroblast differentiation and activation directly or by boosting fibroblast proliferation to increase the myofibroblast precursor pool, leading to fibrosis in the lungs

The expanding knowledge on the pathological features and molecular mechanisms of CNT-induced lung fibrosis is in agreement with the overall understanding of lung fibrosis derived from certain human fibrotic lung diseases and experimental animal models in a number of ways. This correlation suggests that CNT-induced lung fibrosis can be used as a new animal disease model for studying the molecular mechanisms underlying human fibrotic lung diseases, such as IPF and pneumoconiosis. Emerging evidence reveals that CNTs potently induce and activate myofibroblasts both in vitro and in vivo. Moreover, CNTs are found capable of inducing and activating a number of critical mediators and cell signaling pathways that have been implicated in myofibroblast function and regulation during fibrosis development, as summarized in Fig. 5. It is clear that the activation of myofibroblasts likely represents a critical and common molecular step toward the development of organ fibrosis, which now includes CNT-induced lung fibrosis.

Although myofibroblasts have long been recognized as an important group of mesenchymal cells in the development of fibrosis, the contribution of these cells to the fibrotic response induced by fibrogenic particles and fibers, and new materials, such as nanomaterials, has received attention in the field of toxicology only in recent few years. In part, this delayed recognition is due to the difficulty of separating myofibroblasts from fibroblasts during the development of fibrosis with regard to the phenotypes and the available techniques and approaches for analyzing the cells. Indeed, there is a lack of specific markers for identification of myofibroblasts in tissues. Moreover, certain mesenchymal functions during fibrosis are shared between fibroblasts and myofibroblasts, such as the secretion of some ECM proteins. Therefore, it is necessary to use multiple markers, including α-SMA expression, to analyze myofibroblasts and their functions in a specific tissue, time point, and context in which fibrosis takes place. In this respect, the study on CNT-induced lung fibrosis is perhaps advantageous over other lung fibrosis models for the analysis of myofibroblast functions, because the formation of myofibroblasts is exposure-dependent and possibly inducer-specific with respect to the pathological features, mechanisms, and consequences, which can now be readily demonstrated in cultured cells and animals exposed to CNTs.

The fibroblastic response responsible for matrix built-up and scarring is a rather complex and dynamic process, which remains poorly understood to date. From the experimental point of view, the study on myofibroblasts provides an opportunity to unravel the mechanisms underlying the fibroblastic response for the initiation and development of lung fibrosis induced by CNTs, other nanomaterials, and particles and fibers at molecular and cellular levels. From this perspective, the list of mediators and cell signaling pathways summarized above can serve as a reasonable starting point for the mechanistic analysis of myofibroblast regulation and function in fibrosis. Needless to say, further pathological and molecular studies with the aid of genetically engineered mouse strains are required to ascertain the contributions of these candidate mediators and signaling cascades to the onset and pathological outcomes of CNT-induced lung fibrosis in vivo. One caveat to note on this line of research is that these mediators and pathways are not likely to act alone, but work in concert in a highly regulated and time- and context-dependent fashion, to drive the formation and functioning of myofibroblasts leading, ultimately, to fibrosis of the lungs. As such, multiple targets from this myofibroblast-predominant fibroblastic response should be sought after in order to achieve better intervention against lung fibrotic diseases.

Among the identified mediators of myofibroblast activation, TGF-β1 stands out as the most relevant endogenous factor to drive myofibroblast differentiation and function. However, many gaps exist in the understanding of TGF-β1 function and mode of action in the regulation of myofibroblasts and fibrosis induced by CNT exposure. In particular, most toxicological studies on CNT lung fibrosis examined the induction of TGF-β1 mRNA and/or protein expression in cultured cells or in the lungs, which is necessary to establish the involvement of TGF-β1 in CNT toxicity. Several critical questions remain unaddressed. For instance, which signaling pathways and factors mediate the induction of TGF-β1 by CNTs; whether and, if so, how CNTs activate the latent form of TGF-β1 stored in the ECM; and how the activated TGF-β1 controls myofibroblast activation and function upon CNT exposure? The morphologically apparent contractive features of myofibroblasts fittingly explain, at least in part, the inevitable contraction and ultimate scarring of fibrotic tissues in the lungs and other organs. Moreover, the mechano-regulation of matrix remodeling by myofibroblasts appears to be closely correlated with the activation of latent TGF-β1. How this interplay among myofibroblast contraction, tissue stiffness, and TGF-β1 activation occurs to propel CNT-induced lung fibrosis is currently unclear. Apparently, a combination of molecular, biophysical, and genetic approaches is needed to address these questions in future studies.

One implication of the findings from the research on myofibroblasts derives from the notion that organ fibrosis might arise from a failure to suppress the normal repair process of tissue injury to result in the persistent presence and over-functioning of myofibroblasts in fibrotic tissues. It is therefore rational to expect that the information obtained from the study of myofibroblasts and their associated mediators and signaling pathways involved in the pathogenesis of lung fibrosis is likely to generate new insights into both the molecular understanding and the clinical treatment of human fibrotic lung diseases that include IPF, pneumoconiosis, and nanomaterial-induced lung fibrosis.

## Abbreviations

4-HNE: 4-hydroxy-2-nonenal
8-OHdG: 8-hydroxy-2′-deoxyguanosine
Akt: v-akt murine thymoma viral oncogene homolog
BAL: Bronchoalveolar lavage
BMPR2: Bone morphogenetic protein receptor type II
Ccl11: Chemokine (C-C Motif) ligand 11, or eotaxin
CDK: Cyclin-dependent kinase
Chia: Chitinase, acidic, or AMCase
CNT: Carbon nanotube
ECM: Extracellular matrix
ED-A: Extra domain A
EMT: Epithelial-mesenchymal transition
Erk: Extracellular signal-regulated kinase
FoxO3a: Forkhead box O3a
FSP-1: Fibroblast specific protein-1
GSH: Glutathione
GSK-3β: Glycogen synthase kinase-3β
HO-1: Heme oxygenase 1
IL: Interleukin
Il4i1: Interleukin 4 induced 1, or Fig1
IPF: Idiopathic pulmonary fibrosis
JNK: c-Jun N-terminal kinase
Ki-67: marker of proliferation Ki-67
LAP: Latency-associated protein
LLC: Large latency complex
LTBP: Latent TGF-β binding protein
MAPK: Mitogen-activated protein kinase
MEK: Mitogen-activated protein kinase kinase
MMP: Matrix metalloproteinase
MWCNT: Multi-walled carbon nanotube
NADPH: Nicotinamide adenine dinucleotide phosphate
NF-κB: Nuclear factor-κB
NLRP3: Nucleotide-binding oligomerization domain-like receptor: pyrin domain-containing 3
NOX: NADPH oxidase
Nrf2: Nuclear factor erythroid 2-related factor 2
PCNA: Proliferating cell nuclear antigen
PDGF: Platelet-derived growth factor
PDGFR-β: Platelet-derived growth factor receptor-β
PI3K: Phosphoinositide 3-kinase
PP2A: Protein phosphatase 2A
PTEN: Phosphatase and tensin homolog
RNS: Reactive nitrogen species
ROS: Reactive oxygen species
SM22-α: Smooth muscle protein 22-α
Smad: Sma and Mad related family
SPC: Surfactant protein C
SWCNT: Single-walled carbon nanotube
TAK1: TGF-β-activated kinase 1
TGF-β1: Transforming growth factor-β1
Th2: T helper 2
TIMP1: Tissue inhibitor of metalloproteinase 1
TNF-α: Tumor necrosis factor-α
TSP-1: Thrombospondin-1, or Thbs1
VEGF: Vascular endothelial growth factor
Wnt: Wingless-type MMTV integration site family
α-SMA: α-smooth muscle actin
γH2AX: phospho-Histone H2A.X (Ser139)

## Footnotes

1 Dong J and Ma Q. TIMP1 promotes multi-walled carbon nanotube-induced lung fibrosis by stimulating fibroblast activation and p roliferation. 2016; submitted.
